# Klotho overexpression protects human cortical neurons from β-amyloid induced neuronal toxicity

**DOI:** 10.1186/s13041-025-01199-6

**Published:** 2025-03-28

**Authors:** Mohammed R. Shaker, Salam Salloum-Asfar, Rowaida Z. Taha, Ibrahim Javed, Ernst J. Wolvetang

**Affiliations:** 1 Neurological Disorders Research Center, Qatar Biomedical Research Institute, Hamad Bin Khalifa University, Qatar Foundation, Doha, Qatar; 2 College of Health & Life Sciences, Hamad Bin Khalifa University, Qatar Foundation, Doha, Qatar; 3 Australian Institute for Bioengineering and Nanotechnology, The University of Queensland, Brisbane, Qld 4072 Australia; 4 School of Pharmacy and Medical Sciences, UniSA Clinical and Health Sciences, The University of South Australia, Adelaide, SA 5000 Australia

**Keywords:** Klotho, β-amyloid, Neurodegeneration, iPSCs, Cortical neurons, Alzheimer’s disease, Neuroprotection

## Abstract

Klotho, a well-known aging suppressor protein, has been implicated in neuroprotection and the regulation of neuronal senescence. While previous studies have demonstrated its anti-aging properties in human brain organoids, its potential to mitigate neurodegenerative processes triggered by β-amyloid remains underexplored. In this study, we utilised human induced pluripotent stem cells (iPSCs) engineered with a doxycycline-inducible system to overexpress *KLOTHO* and generated 2D cortical neuron cultures from these cells. These neurons were next exposed to pre-aggregated β-amyloid 1–42 oligomers to model the neurotoxicity associated with Alzheimer’s disease. Our data reveal that upregulation of *KLOTHO* significantly reduced β-amyloid-induced neuronal degeneration and apoptosis, as evidenced by decreased cleaved caspase-3 expression and preservation of axonal integrity. Additionally, *KLOTHO* overexpression prevented the loss of dendritic branching and mitigated reductions in axonal diameter, hallmark features of neurodegenerative pathology. These results highlight Klotho’s protective role against β-amyloid-induced neurotoxicity in human cortical neurons and suggest that its age-related decline may contribute to neurodegenerative diseases such as Alzheimer’s disease. Our findings underscore the therapeutic potential of Klotho-based interventions in mitigating age-associated neurodegenerative processes.

**Supplementary Information:**

The online version contains supplementary material available at 10.1186/s13041-025-01199-6.

Klotho, widely recognized as an aging suppressor gene, plays a critical role in modulating aging processes and maintaining cognitive function. Mutations in the 5′ flanking region of its alpha subtype are associated with premature aging, cognitive decline, and reduced lifespan in mice [1]. The *KLOTHO* gene, located on human chromosome 13, comprises five exons and four introns in its coding region [2]. The full-length alpha form of KLOTHO undergoes cleavage by α- and β-secretases, yielding a soluble form of αKLOTHO. Alternatively, splicing of *αKLOTHO* RNA generates a secreted form of the protein [3]. Both soluble and secreted forms circulate systemically, functioning as hormones and co-receptors, notably in conjunction with fibroblast growth factor (FGF) [4].

The levels of αKLOTHO diminish with age, beginning around the fourth decade of life in humans [5]. Within the central nervous system, *klotho* deficiency has been linked to a reduction of synapse numbers, disruptions in axonal transport, neuronal degeneration, and impaired myelin production [6, 7]. Our previous work demonstrated that overexpression of *KLOTHO* inhibits neuronal senescence in human cellular models, highlighting its potential therapeutic significance in combating neuronal aging and degeneration [8]. Conversely, individuals that carry mutations that result in increased levels of klotho are protected from Alzheimer’s disease and cognitive decline [9].

Various transgenic animal models and mammalian in vitro cellular assays have been developed to replicate aging signatures in the laboratory [10, 11], as aging remains the primary risk factor for neurodegenerative diseases, including Alzheimer’s disease [12]. iPSC-derived neurons offer a robust platform for disease modelling and drug screening applications, enabling initial risk assessments [13, 14]. Notably, iPSC-derived neurons have been extensively employed to study human cellular aging and to model late-onset neurodegenerative diseases [11]. For instance, artificially aged neurons can be generated via ectopic expression of progerin, which induces hallmarks of cellular aging in neurons [15].

Prolonged culture of iPSC-derived neurons in vitro introduces cellular stress, elevating markers of neuronal aging, such as increased senescence associated beta-galactosidase activity, telomere shortening, and neuronal degeneration [8]. Cleavage of amyloid precursor protein (APP) produces a range of Aβ peptides, ranging from 36 to 43 amino acids, with the longer Aβ peptide, Aβ_42_, demonstrating a higher propensity to aggregate and form the characteristic plaques observed in patients with Alzheimer’s disease [16]. The neurotoxicity of Aβ originates from the oligomeric form which is an intermediate and transient stage in the fibrilization paradigm of Aβ. Thus, exposure of human cortical neurons to pre-aggregated Aβ_42_ is a widely used approach to assess its effects on neuronal health, viability, morphology, synaptic integrity, and mitochondrial function [11].

Previously, Klotho was implicated in the sequestration and transport of β-amyloid in murine brain cells [17]. Having established that KLOTHO prevents the increase of several hallmarks of senescence observed in Alzheimer’s disease [18], we here sought to determine whether *KLOTHO* provides resistance to β-amyloid-induced neuronal degeneration and death. To address this, we utilised a recently published human dCas9-VPR *KLOTHO*-inducible iPSC line [8]. These iPSCs were engineered with a doxycycline (dox)-inducible dCas9-VPR cassette targeted at the AAVS1 safe harbor site. This system enables dox-dependent upregulation of a target gene, following co-delivery of gRNAs directing the VPR transcriptional activator to the gene’s promoter while preserving cell type-specific splicing [8].

Using dCas9-VPR *KLOTHO*-inducible iPSC system, we generated neural progenitors (NPCs)-cortical neurons which were differentiated over overtime (Fig. 1A and B). qPCR analysis of *EMX2* and *OTX2* confirmed the forebrain identity of these neurons (Figure S1A), while *PAX6*, *DCX*, and *CTIP2* analysis showed the successful differentiation of NPCs towards cortical neurons (Figure S1A). Interestingly, immunocytochemistry revealed predominant expression of KLOTHO in axons (Fig. 1B).

**Fig. 1:**
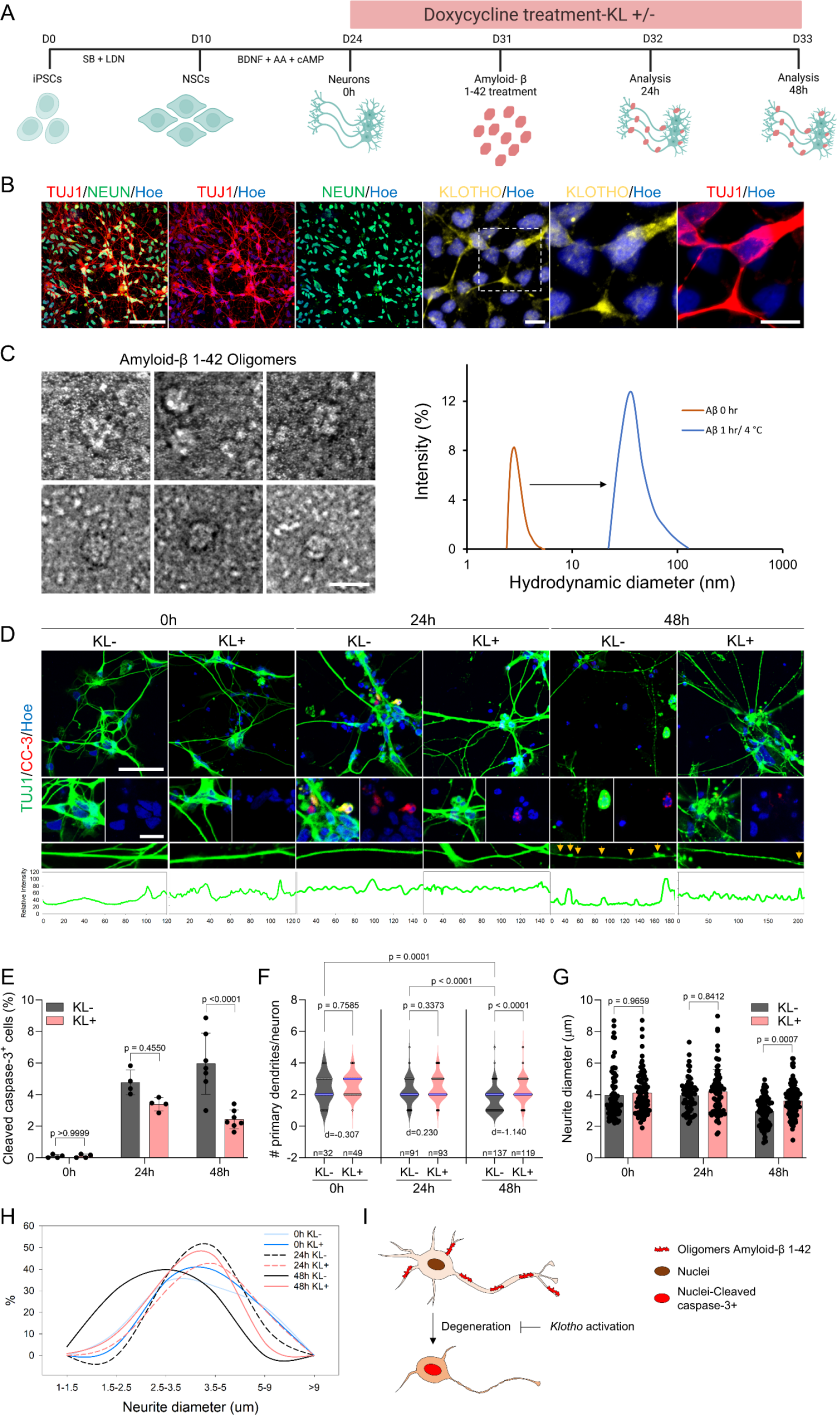
Klotho upregulation protects human neurons from β-amyloid-induced toxicity. (**A**) Schematic overview of the protocol for generating mature human neurons from dCas9-VPR iPSCs transduced with 3 gRNAs. Neurons were pretreated with doxycycline (dox) for 1 week to induce *KLOTHO* expression (KL+), followed by exposure to 5µM β-amyloid for 24–48 h Created with BioRender.com. (**B**) Immunostaining of iPSC-derived neurons with TUJ1 (Red), NEUN (Green), and KLOTHO (Yellow). Nuclei were counterstained with Hoechst 33,342 (Blue). Zoomed images show distinct KLOTHO expression in neuronal axons. Scale bar = 60 μm; zoomed images scale bar = 10 μm. (**C**) Liquid-phase transmission electron microscopy images of small and large oligomers of β-amyloid 1–42 pre-aggregated for 1 h at 4 °C. Scale bar = 10 nm. Right graph shows the size distribution of β-amyloid monomers (orange) and oligomers (blue) measure by DLS at 4 °C. (**D**) Cultured neurons pretreated with and without dox, and exposed to β-amyloid aggregates for 24 h and 48 h. Immunostaining of with TUJ1 (Green) and CLEAVED CASPASE-3 (CC3, Red) after treatment with β-amyloid for 24–48 h. Yellow arrows indicate TUJ1 puncta along neurites. Scale bar = 50 μm; zoomed images scale bar = 10 μm. (**E**) Quantification of the percentage of neurons expressing CLEAVED CASPASE-3. Data are presented as mean ± standard deviation; p values were measured via One Way ANOVA with Tukey’s multiple comparisons test; *n* = 4 independent experiments. (**F**) Violin plots showing the number of primary neurites per neuron in cultures with (KL+) and without (KL-) *KLOTHO* upregulation exposed to β-amyloid for 0, 24, and 48 h. p values were measured via One Way ANOVA with Tukey’s multiple comparisons test; *n* = 3 independent experiments; total number of analysed neurons = 565 neurons; d indicates Cohen’s d analysis. (**G**) Quantification of neurite diameter in neurons exposed to β-amyloid with or without *KLOTHO* upregulation at 0, 24, and 48 h. Data are presented as mean ± standard deviation; p values were measured via One Way ANOVA with Tukey’s multiple comparisons test; *n* = 3 independent experiments; total number of analysed neurons = 515 neurons. (**H**) Distribution of neurite diameters in neurons with (KL+) and without (KL-) *KLOTHO* upregulation at 0, 24, and 48 h of β-amyloid exposure. (**I**) Schematic summary illustrating Klotho’s neuroprotective role in mitigating β-amyloid-induced toxicity in human iPSC-derived neurons. KLOTHO upregulation preserves neurite integrity and reduces apoptotic signalling

To investigate the neuroprotective effects of KLOTHO, we treated neurons with 1 µg/ml dox for one week to induce *KLOTHO* expression, and next challenged these neurons for three days with 5 µM pre-aggregated β-amyloid 1–42 (Fig. 1A). Transmission electron microscopy confirmed the formation of oligomers of β-amyloid 1–42 with blobs of protein in circular or disk like morphology (Fig. 1C, S1B). The hydrodynamic radius of β-amyloid monomers was determined to be 2.7 ± 1.2 nm, whereas that of oligomers was 36.4 ± 8.2 nm, indicating increased morphological heterogeneity in β-amyloid aggregates (Fig. 1C). The concentration of purified β-amyloid oligomers following separation from monomers was quantified as 4.1 ± 0.8 µM (Fig. 1C). Neuronal cell death was assessed over time, showing that β-amyloid treatment induced neuronal death in a time-dependent manner, as indicated by an increased percentage of cleaved caspase-3-positive neurons at 24 and 48 h compared to untreated neurons (Fig. 1D and E). Importantly, upregulation of *KLOTHO* prior to β-amyloid oligomers exposure significantly inhibited β-amyloid neurotoxicity and prevented axonal degeneration (Figs. 1D, yellow arrows, 1E).

Given that dendritic branching is strongly associated with neurodegeneration and synaptic connectivity, we quantified the number of dendritic branches. Exposure to β-amyloid oligomers resulted in a significant reduction in primary dendrite branching (Fig. 1F), and *Klotho* overexpression mitigated this loss and preserved dendritic integrity (Fig. 1F; Table S3). We applied Cohen’s d analysis to quantify the differences between the two groups, the data of Cohen’s d analysis showed greater differences (d = -1.140) between *KLOTHO* overexpression and the untreated neurons at 48 h indicating that KLOTHO overexpression significantly protected neurons from dendritic degeneration caused by β-amyloid toxicity over time (Fig. 1F).

Additionally, we observed a reduction in neurite diameter following β-amyloid oligomers exposure, with significant axonal degeneration occurring by 48 h (Figs. 1D, yellow arrows, 1G, 1 H). Again, neurons with *KLOTHO* overexpression exhibited a smaller reduction in axonal diameter compared to those without *KLOTHO* overexpression (Fig. 1G H). However, despite the protective effects, *KLOTHO* overexpression did not fully sustain axonal health to levels comparable to 0 and 24 h of β-amyloid exposure at the 48-hour time point (Fig. 1D and G).

Collectively, these findings demonstrate that KLOTHO expression protects human iPSC-derived cortical neurons from β-amyloid oligomers-induced toxicity. Our results highlight the direct neuroprotective effects of KLOTHO, underscoring its potential to improve brain function during aging and supporting its promise as a therapeutic target for age-related neurodegenerative diseases such as Alzheimer’s disease.

Animal studies demonstrated that overexpression of Klotho enhances myelination [19], synaptic plasticity, and cognitive functions [20, 21]. In our previous work, we showed that overexpression of KLOTHO inhibits neuronal senescence in human brain organoids [8], highlighting its potential as a potent anti-aging factor that protects against neurodegeneration. In this study, we investigated to what extent *KLOTHO* is able to protect human cortical neurons from β-amyloid 1–42 oligomers-induced neurodegeneration.

Our findings reveal that Klotho is predominantly expressed in the axons of cortical neurons, consistent with our earlier observations [8] and those of others [22, 23]. Importantly, the endogenous upregulation of *KLOTHO* was sufficient to delay neuronal degeneration caused by oligomers of β-amyloid 1–42. While this study did not address the long-term effects of β-amyloid 1–42 oligomers exposure, we speculate that prolonged exposure would eventually lead to complete cortical neuron degeneration and death. This conclusion is supported by our observation of a substantial reduction in axonal diameter in neurons treated with β-amyloid 1–42 oligomers, even with *KLOTHO* upregulation.

Accumulation of β-amyloid at pre- and post-synaptic sites has been detected in Alzheimer’s disease, contributing to synaptic dysfunction and neuronal loss [24]. With ageing, β-amyloid levels increase, forming oligomers that induce the formation of membrane pores, causing excessive calcium influx and further neurotoxicity [25]. Notably, Klotho overexpression has been shown to enhance the function of NMDA receptors, particularly the GluN2B subunit, which is crucial for synaptic plasticity and neuronal survival [26]. NMDA receptor activation boosts antioxidant defense through the thioredoxin/peroxiredoxin (Trx/Prx) system, mitigating β-amyloid neurotoxicity [20]. Given that Klotho enhances Trx/Prx-mediated neuroprotection, and our data show robust expression of Klotho in cortical neurons, it is likely that Klotho exerts its protective effects by stabilizing NMDA receptor activity, counteracting β-amyloid-induced calcium dysregulation, and reinforcing antioxidant defense. Future studies should explore these mechanistic aspects in both in vitro and in vivo models to provide deeper insights into Klotho’s therapeutic potential for Alzheimer’s disease.

While this study primarily focused on the acute effects of β-amyloid oligomers on cortical neurons, Alzheimer’s disease is characterised by chronic β-amyloid accumulation [27], which drives tau pathology and ultimately leads to widespread neuronal loss. While our findings demonstrate that KLOTHO plays a protective role against acute β-amyloid-induced neuronal toxicity, further studies are needed to explore the long-term impact of KLOTHO overexpression in models that better recapitulate chronic β-amyloid accumulation and its downstream pathological effects.

Given the rising incidence of Alzheimer’s disease, various preclinical models have been developed to study Alzheimer’s disease pathophysiology, including transgenic mouse models carrying Alzheimer’s disease-associated mutations that lead to increased β-amyloid accumulation [28]. Despite extensive research, clinical trials targeting β-amyloid reduction, either by preventing its formation or clearing plaques, have yet to yield significant cognitive improvements in human patients [29]. Notably, previous studies have demonstrated that low-dose systemic administration of Klotho enhances cognitive function in animal models [30]. To further evaluate the therapeutic potential of Klotho in Alzheimer’s disease, future studies should investigate the effects of both low- and high-dose Klotho administration in transgenic mouse models of Alzheimer’s disease. These studies will provide critical insights into whether Klotho can mitigate β-amyloid-driven neurodegeneration and cognitive decline in a chronic disease setting.

Nonetheless, our data demonstrate the potent anti-degenerative effects of KLOTHO in mitigating β-amyloid-induced neuronal toxicity. These findings support the hypothesis that the age-related decline in Klotho expression may be a significant contributing factor to neurodegenerative diseases such as Alzheimer’s disease. By elucidating the neuroprotective role of KLOTHO, this study provides a basis for further exploration of KLOTHO-based therapeutic strategies to combat ageing-related neurodegeneration.

## Materials and methods

### Human embryonic stem cells culture and cortical organoids generation

Human embryonic stem cells (H9, WiCell Research Institute, WA09 cells) and WTC iPSC lines (a gift from Professor Bruce Conklin) were cultured on Matrigel-coated plates (StemCell Technologies, Cat. #354277) in mTeSR™ Plus medium (StemCell Technologies, Cat. #100–0276) as previously described [31].

### Generation of human neurons

iPSCs and ESCs were maintained in feeder-free conditions using mTeSR™ Plus medium. Neural progenitor differentiation was induced as previously described [32, 33]. Briefly, mTeSR™ Plus medium was replaced with N2 medium containing DMEM/F12 (Gibco, Cat. #11320-33), 2% B-27 supplement (Gibco, Cat. #17504044), 1% N-2 supplement (Gibco, Cat. #17502-048), 1% MEM Non-Essential Amino Acids (Gibco, Cat. #11140-050), 1% penicillin/streptomycin (Gibco, Cat. #15140148), and 0.1% β-mercaptoethanol (Gibco, Cat. #21985-023). Cells were treated daily with dual SMAD inhibitors (10 µM SB-431542, Miltenyi Biotec, Cat. #130-106-543, and 0.1 µM LDN-193189, Stemgent, Cat. #04–0074) for 10 days with daily medium changes. At day 11, neural progenitors were detached using Accutase (Gibco, Cat. #A11105-01) and seeded as single cells onto 18-mm coverslips coated with Poly-L-ornithine (Sigma, Cat. #P4957) and Laminin (5 mg/mL; Thermo Fisher, Cat. #23017015) in the presence of basic fibroblast growth factor (bFGF, 20 ng/mL; R&D, Cat. #233-FB-01 M) for 12 h. Neuronal differentiation was induced by replacing N2 medium with Neurobasal medium (Gibco, Cat. #A35829-01) containing 2% B-27, 1% N-2, 1% penicillin/streptomycin, 0.025% Insulin (Sigma, Cat. #I9278), 10 ng/mL BDNF (Lonza-Peprotech, Cat. #450-02-50), 0.2 µg/mL L-Ascorbic acid (Sigma, Cat. #A4544), and 0.1 mM cAMP (Sigma, Cat. #D0627). Neurons were differentiated for two weeks, then fixed in 4% paraformaldehyde (PFA, Thermo Fisher, Cat. #ALF043368.9 M) for 10 min at room temperature and immunostained with neuronal markers. For β-amyloid treatment, β-amyloid Protein Fragment 1–42 (Sigma, Cat. #A9810) was prepared at a stock concentration of 5 mM in distilled water. Oligomeric preparations were achieved by diluting β-amyloid and incubating it for 1 h at 4 °C before adding it to neurons at a final concentration of 5 µM for 24–48 h. The hydrodynamic diameter of β-amyloid monomers and oligomers was measured by dynamic light scattering (DLS) under ambient conditions using a Malvern Instruments system, following established protocols [34]. Oligomers were purified from monomers by ultrafiltration using 10 kDa molecular weight cutoff (MWCO) spin filters (Millipore) and subsequently washed three times with deionized water. The final oligomer concentration was determined using the Pierce BCA protein assay kit (Thermo Fisher Scientific).

### Immunocytochemistry

Immunocytochemistry (ICC) were performed as described previously [35]. In brief, cells were washed three times with PBS for 10 min at room temperature, followed by blocking in 3% bovine serum albumin (Sigma, Cat. #A9418-50G) and 0.1% Triton X-100 in PBS for 1 h. Primary antibodies were applied overnight at 4 °C, followed by three washes with PBS. Samples were incubated with Alexa Fluor-conjugated secondary antibodies (Jackson ImmunoResearch Laboratory) for 1 h at room temperature, counterstained with Hoechst 33,342 (Invitrogen, Cat. #H3570), and mounted for imaging. Images were acquired using a Leica TCS SP8 confocal microscope housed in the SBMS Imaging Facilities at the University of Queensland. A list of primary antibodies is provided in Table S1.

### RNA extraction and RT-qPCR

Total RNA was extracted from neuronal precursor cells (NPCs) and cortical mature neurons at week 6 (W6) and week 8 (W8) (*N* = 3 per condition) using the Direct-zol RNA Micro Prep Extraction Kit (Zymo Research) following the manufacturer’s protocol. RNA concentration and purity were assessed using a NanoDrop spectrophotometer. Complementary DNA (cDNA) was synthesized from total RNA using the Superscript IV Reverse Transcriptase Kit (Thermo Fisher Scientific) according to the manufacturer’s instructions. RT-qPCR was performed to quantify the expression of regional molecular markers, NPC markers, and neuronal markers. Specific primers for EMX2, OTX2, FOXA1, PAX6, MAP2, DCX, CTIP2 and SATB2 were used (sequences provided in Table S2). The reaction mixture, prepared with a PowerUp SYBR Green Master Mix (Thermo Fisher Scientific), was loaded into a MicroAmp Fast Optical 96-Well Reaction Plate. Thermal cycling was performed on an Applied Biosystems 7500 Fast Real-Time PCR System under the following conditions: initial denaturation at 95 °C for 2 min, followed by 40 cycles of 95 °C for 15 s and 60 °C for 1 min. All reactions were run in triplicates. Gene expression was normalized to GAPDH, and relative quantification was determined using the ΔΔCt method.

### Transmission electron microscopy

β-amyloid oligomers (5 μm, 10 uL) solution was placed on a glow-discharged carbon-coated copper grid. The sample was blotted after 30 s and grid was washed with deionised water by blotting with a filter paper. The grid was negatively stained with 1% uranyl acetate solution for 20 s and washed with droplet of water twice by blotting with filter paper. The sample was imaged with a Hitatchi HT7700 transmission electron microscopy operating at 120 kV.

### Statistical analysis

Statistical analyses were performed using GraphPad Prism 9 as recently described [36]. Data are expressed as mean ± standard deviation for normally distributed data or as median ± standard deviation for non-normally distributed data. Sample sizes were determined using power analysis and are detailed in figure legend. Comparisons between two groups were analyzed using Student’s t-tests, while one-way or two-way ANOVA followed by Tukey’s post-hoc tests were used for multiple group comparisons. Statistical significance was defined as *P* < 0.05.

## Electronic supplementary material

Below is the link to the electronic supplementary material.

Figure S1. Analysis of Cortical Neurons and Electron Microscopy of β-Amyloid Oligomers. (A) Representative graphs showing qPCR analysis of the expression levels of markers of regional (EMX2, OTX2), NPCs (PAX6), immature neurons (DCX), and mature cortical neurons (CTIP2) in NPCs and differentiated cortical neurons at two timepoints: week 6 (W6) and week 8 (W8). Relative gene expression was normalized to GAPDH. Data are presented as mean ± standard deviation; p values was determined using ordinary one-way ANOVA with Dunnett's multiple comparisons test. Results represent triplicate experiments. (B) Transmission electron microscopy images of β-amyloid oligomers prepared at 5 µM for 1 hour at 4°C. Scale bars = 50 nm, and 200 nm

Supplementary Material 2

Table S3. Raw data of number of primary neurites quantified per neuron

## Data Availability

The data that support the findings of this study are available from the corresponding author upon reasonable request.
