# Supplementary material for: Klotho overexpression protects human cortical neurons from β-amyloid induced neuronal toxicity
Source: Mol Brain. 2025 Mar 28;18:27. doi: 10.1186/s13041-025-01199-6 (PMC11954210; doi:10.1186/s13041-025-01199-6)
Supplement: Supplementary file 2 — Supplementary Material 2 [file 13041_2025_1199_MOESM2_ESM.docx]

**Table S1. List of Antibodies used for immunohistochemistry**

| **Antigen** | **Host Species** | **Source** | **Cat#** | **Dilution** |
| --- | --- | --- | --- | --- |
| KLOTHO | Rabbit | Bioss | bs-2925R | 1:500 |
| TUJ1 | Rabbit | Cell Signaling | D71G9 | 1:1000 |
| TUJ1 | Mouse | Sigma | T8578 | 1:1000 |
| NEUN | Mouse | Millipore | MAB377 | 1:500 |
| NEUN | Rabbit | Millipore | ABN78 | 1:500 |
| CLEAVED CASPASE-3 | Rabbit | Cell Signaling | 9661 | 1:500 |

**Table S2. List of Primer Sequences used for qPCR (5′−3′ orientation).**

| **Gene** | **Primers** |
| --- | --- |
| *EMX2*- Forward | GCTTCTAAGGCTGGAACACG |
| *EMX2*-Reverse | TTGCGAATCTGAGCCTTCTT |
| *DCX-*Forward | CCTTGGCTAGCAGCAACAGT |
| *DCX*-Reverse | CCACTGCGGATGATGGTAA |
| *OTX2-* Forward | GCTGTAAGTTCCACTGCTC |
| *OTX2-* Reverse | GCTGTAAGTTCCACTGCTC |
| *CTIP2*-Forward | TGGGTGCCTGCTATGACAAG |
| *CTIP2*-Reverse | GGCTCGGACACTTTCCTGAG |
| *PAX6-*Forward | ATGTGTGAGTAAAATTCTGGGCA |
| *PAX6*-Reverse | GCTTACAACTTCTGGAGTCGCTA |
| *GAPDH*-Forward | CATGAGAAGTATGACAACAGCCT |
| *GAPDH*-Reverse | AGTCCTTCCACGATACCAAAGT |
